# Adipocyte Model of Mycobacterium tuberculosis Infection Reveals Differential Availability of Iron to Bacilli in the Lipid-Rich Caseous Environment

**DOI:** 10.1128/IAI.00041-18

**Published:** 2018-05-22

**Authors:** Ananya Nandy, Anupam Kumar Mondal, Rajesh Pandey, Prabhakar Arumugam, Stanzin Dawa, Neetika Jaisinghani, Vivek Rao, Debasis Dash, Sheetal Gandotra

**Affiliations:** a CSIR-Institute of Genomics and Integrative Biology, New Delhi, India; b Academy of Scientific and Innovative Research, Ghaziabad, India; c CSIR Ayurgenomics Unit—TRISUTRA, CSIR-Institute of Genomics and Integrative Biology, New Delhi, India; Weill Cornell Medical College

**Keywords:** Mycobacterium tuberculosis, adipocyte, granuloma, host-pathogen interactions, lipid metabolism

## Abstract

Mycobacterium tuberculosis, a successful human pathogen, utilizes multiple carbon sources from the host but adapts to a fatty-acid-rich environment *in vivo*. We sought to delineate the physiologic response of M. tuberculosis to a lipid-rich environment by using differentiated adipocytes as a model system. Global transcriptome profiling based on RNA sequencing was performed for bacilli from infected adipocytes and preadipocytes. Genes involved in *de novo* fatty acid synthesis were downregulated, while those predicted to be involved in triglyceride biosynthesis were upregulated, in bacilli isolated from adipocytes, indicating reliance on host-derived fatty acids. Transcription factor network analysis indicated suppression of IdeR-regulated genes, suggesting decreased iron uptake by M. tuberculosis in the adipocyte model. This suppression of iron uptake coincided with higher ferritin and iron levels in adipocytes than in preadipocytes. In accord with the role of iron in mediating oxidative stress, we observed upregulation of genes involved in mitigating oxidative stress in M. tuberculosis isolated from adipocytes. We provide evidence that oleic acid, a major host-derived fatty acid, helps reduce the bacterial cytoplasm, thereby providing a safe haven for an M. tuberculosis mutant that is sensitive to iron-mediated oxidative stress. Via an independent mechanism, host ferritin is also able to rescue the growth of this mutant. Our work highlights the inherent synergy between macronutrients and micronutrients of the host environment that converge to provide resilience to the pathogen. This complex synergy afforded by the adipocyte model of infection will aid in the identification of genes required by M. tuberculosis in a caseous host environment.

## INTRODUCTION

Transcriptional profiling of pathogens in relevant host environments has led to increased understanding of the host-pathogen dialogue and to the identification of pathways that are essential for pathogen survival in a niche-specific manner. Several studies have indicated that the human pathogen Mycobacterium tuberculosis adapts toward the utilization of lipids *in vivo* (1–4). The relevance of understanding the physiology of the pathogen in a lipid-rich environment is further supported by the fact that M. tuberculosis resides in triglyceride-rich caseating granulomas in tuberculosis patients (5, 6). These granulomas constitute a complex environment of extracellular bacilli that emerge after the necrosis of infected cells (7).

There have been several attempts to delineate the physiology of M. tuberculosis in a lipid-rich environment using transcriptome analysis, largely limited to *in vitro* conditions of fatty acids as the chief carbon source or the addition of detergents that mimic fatty acid stress (8, 9). However, M. tuberculosis is known to cocatabolize multiple carbon sources (10). The problem is compounded by the fact that nutrient availability in the host may affect other physiological characteristics that affect innate immune defenses (11). For example, as macrophages differentiate to foamy macrophages in atherosclerosis, in addition to the lipid-loaded phenotype, lysosomal acidification is impaired (12). The differentiation process may bring about differences in the relative abundances of several macro- and micronutrients that cannot be envisaged in a simplified *in vitro* model. Therefore, a complex cellular system with lipids as the chief variable is required in order to understand pathogen physiology in a lipid-rich host environment.

To understand the relevance of host lipid-associated features in guiding the adaptation of M. tuberculosis to a lipid-rich environment, we used an adipocyte model of infection. Here we found bacilli achieving growth in a lipid-rich necrotic environment offered by adipocytes during infection. We used RNA sequencing (RNA-seq) to perform global transcriptome profiling of M. tuberculosis isolated from adipocytes and preadipocytes. Physiological and biochemical evidence supported transcriptomic evidence on increased fatty acid and differential iron availability encountered by bacilli associated with adipocytes, with the bacilli acquiring an increased ability to mitigate oxidative stress. This work led us to identify an unprecedented link between iron storage in bacteria and the lipid content of the host cell. This finding provides a new perspective on host-pathogen interactions and raises the possibility that microbes that invade lipid-rich niches may have an inherent robust capacity to counter oxidative stress; hence, targeting these may provide improved pathogen clearance.

## RESULTS

### Adipocyte infection offers a model for the study of M. tuberculosis physiology in a caseous necrotic environment.

**FIG 1:**
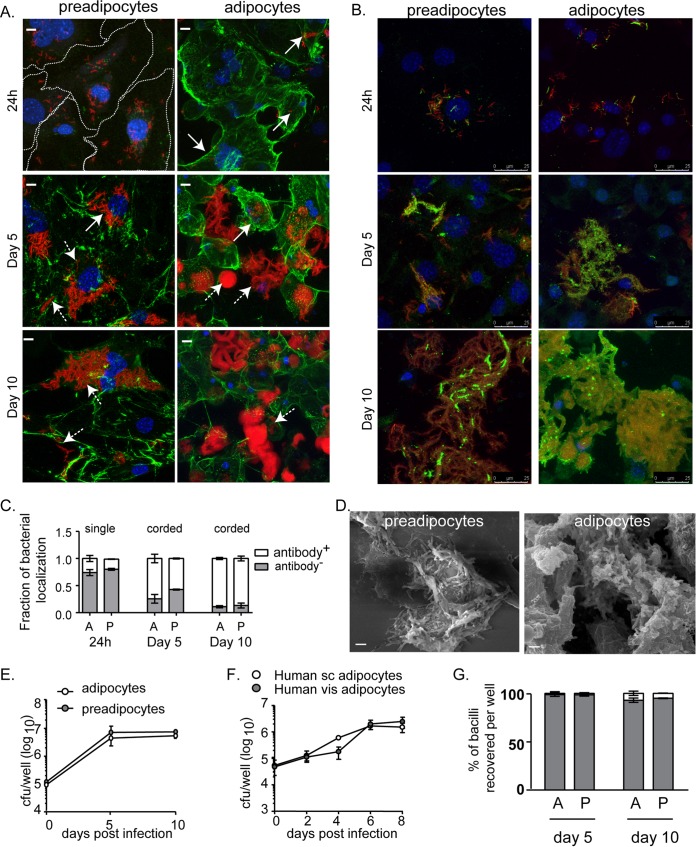
Adipocytes and preadipocytes provide a growth-permissive environment for M. tuberculosis H37Rv. (A and B) Maximum-intensity projection of a confocal z-stack of 3T3L1 preadipocytes and adipocytes infected with H37Rv-mCherry (red) for the indicated time points. Cell surfaces were labeled by immunostaining for collagen (A) and M. tuberculosis lipoarabinomannan (B) (green). Nuclei were stained with DAPI (blue). Extracellular and intracellular bacilli are indicated by dashed- and solid-line arrows, respectively. Bars in panel A, 10 μm. (C) Quantification of M. tuberculosis LAM-specific staining. Data for 24-h infection (left) represent single bacteria (∼350), while those for day 5 (center) and day 10 (right) are from cords (∼130), since no single bacilli were observed. Data are means ± standard errors of the means for three independent experiments. A, adipocytes; P, preadipocytes. (D) Scanning electron micrographs of infected cells at day 10. Bars, 2 μm. (E). Growth kinetics of H37Rv in 3T3L1 preadipocytes and adipocytes. Data are means ± standard deviations for three wells and represent the results of five experiments. (F) Growth kinetics of H37Rv in adipocytes derived from human subcutaneous (sc) and visceral (vis) fibroblasts. Data are means ± standard deviations for three wells. (G) Percentages of bacilli (CFU) per well isolated from the cell lysate (shaded) and medium (open) fractions at the indicated time points after infection of adipocytes and preadipocytes. Data are means ± standard deviations for three wells and represent the results of five experiments.

Transcriptional signatures of M. tuberculosis in human sputum and granulomas (13, 14) are limited in their ability to highlight the heterogeneity in bacterial physiology based on the lesion environment. We wanted to understand the physiology of M. tuberculosis in a lipid-rich cellular environment, and therefore, we utilized triglyceride-rich 3T3L1 adipocytes and their precursor preadipocytes as hosts for infection. Preadipocytes were differentiated to adipocytes with concomitant increases in triglyceride pools (see Fig. S1A in the supplemental material). To check if adipocytes and preadipocytes internalize M. tuberculosis, we immunostained for collagen or the M. tuberculosis surface, followed by confocal microscopy (Fig. 1A, B, and C). At 24 h, ∼80% of bacilli were intracellular (Fig. 1C). However, as the duration of infection increased, there was evident extracellular growth of bacteria and necrosis of cells (Fig. 1; also Fig. S1B in the supplemental material). At this point, both cell types lost their integrity, and mycobacterial cords were found to be extracellular and tightly bound to the necrotic cells (Fig. 1B, C, and D). Bacillary counts increased almost 50-fold in 5 days and remained constant thereafter in both cellular models of infection (Fig. 1E). The sustenance of bacillary growth by primary human adipocytes corroborated the growth of H37Rv on 3T3L1 adipocytes (Fig. 1F). By determining CFU counts in the culture supernatants and surface-attached contents of the well separately, we observed that the majority of the bacilli grew attached to the necrotic cells, while only ≤9% of the total population was recoverable from the cell culture medium (Fig. 1G). There was no difference in the number of surface-attached bacteria or the medium-recoverable fraction of bacteria between adipocytes and preadipocytes (Fig. 1G). We utilized the cell-impermeant DNA dye Sytox green to measure necrosis. As early as 24 h postinfection, we observed approximately 5% Sytox green-positive adipocytes and preadipocytes; this proportion increased over 8 days to 50% and 20%, respectively (Fig. S1B). By day 10 postinfection, very few 4′,6-diamidino-2-phenylindole (DAPI)-positive nuclei could be observed (Fig. 1A and B). Therefore, we concluded that both the cellular models of infection provide necrotic cellular environments, one with higher triglycerides than the other. The surface-attached bacteria from both adipocytes and preadipocytes were further studied on the hypothesis that the necrotic adipocyte environment would mimic the necrotic lipid-rich environment of caseous granulomas found in human tuberculosis, whereas the preadipocyte environment, although necrotic, would not be lipid rich and therefore could serve as a control.

### Global transcriptional profiling of M. tuberculosis in adipocytes and preadipocytes.

**FIG 2:**
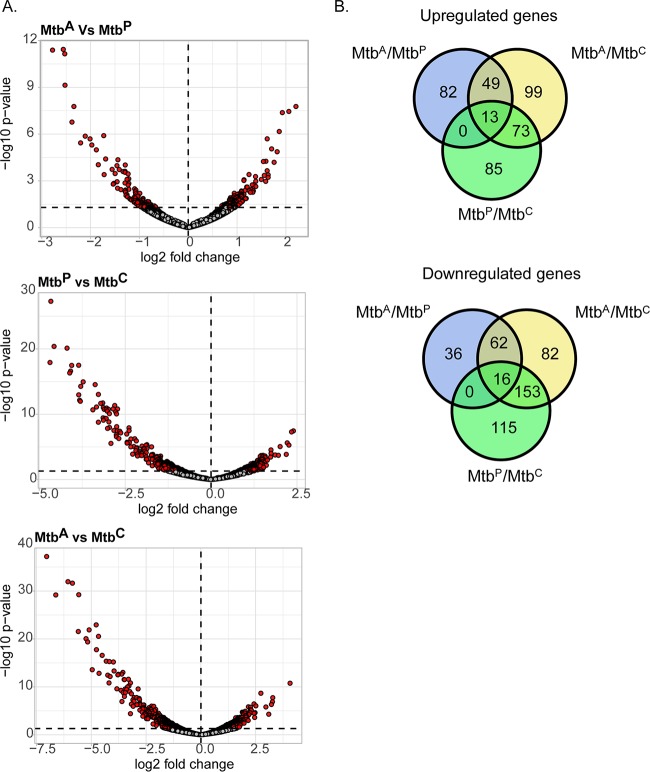
RNA sequencing and analysis of differential gene expression of M. tuberculosis in adipocytes. (A) Volcano plots showing differentially expressed genes (*P* < 0.05) (red circles) of M. tuberculosis grown in each pairwise comparison. (B) Venn diagrams showing the numbers of genes that are common or mutually exclusive across all pairwise comparisons.

We performed RNA-seq on M. tuberculosis isolated 10 days postinfection from adipocytes (Mtb^A^), preadipocytes (Mtb^P^), and complete DMEM (Dulbecco's modified Eagle medium), used as a medium control (Mtb^C^). The RNA was subjected to sequencing, followed by analysis for differentially expressed genes (at a *P* value of <0.05). We combined data from two biological replicates, with which we obtained 7 million and 7.9 million reads from Mtb^A^, 7.8 million and 6.5 million reads from Mtb^P^, and 5.5 million and 8.7 million reads from Mtb^C^ (Table 1). We found 144 protein-coding genes to be upregulated and 114 protein-coding genes to be downregulated in Mtb^A^ relative to Mtb^P^ (Fig. 2; see also File S1 in the supplemental material). Of the genes upregulated in Mtb^A^ relative to Mtb^P^, 34% were also upregulated in Mtb^A^ relative to Mtb^C^ but not in Mtb^P^ relative to Mtb^C^, while 54% of the genes downregulated in Mtb^A^ relative to Mtb^P^ were also downregulated in Mtb^A^ relative to Mtb^C^ but not in Mtb^P^ relative to Mtb^C^ (Fig. 2B; see also File S2 in the supplemental material). Therefore, we found a significant proportion of differential gene expression to be specific to the adipocyte environment rather than simply infection driven.

**TABLE 1:** RNA-seq library characteristics for two experiments and three conditions^*a*^

Sample	Total reads	% alignment
Mtb^A^−1	7,000,987	83.46
Mtb^A^−2	7,903,126	80.81
Mtb^P^−1	7,800,460	86.83
Mtb^P^−2	6,488,427	73.06
Mtb^C^−1	5,521,296	84.11
Mtb^C^−2	8,756,349	83.01

a M. tuberculosis isolated from adipocytes (Mtb^A^), preadipocytes (Mtb^P^), or DMEM (Mtb^C^) was tested in experiments 1 and 2.

### M. tuberculosis accumulates triglyceride by utilizing host-derived fatty acids.

**FIG 3:**
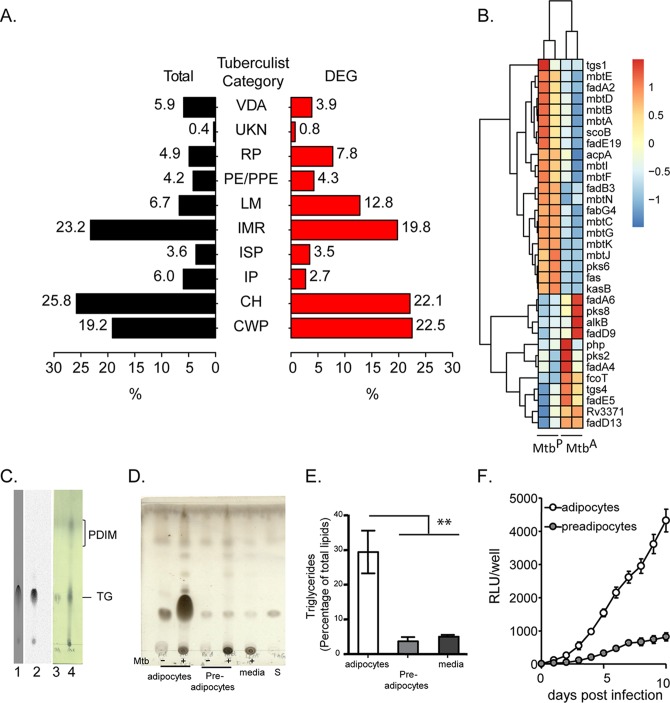
M. tuberculosis utilizes host-derived fatty acid for storage and oxidative metabolism in adipocytes. (A) Classification (based on TubercuList) of differentially expressed (DEG) versus total genes of M. tuberculosis. Gene categories are as follows: VDA, virulence, detoxification, adaptation; UKN, unknown; RP, regulatory proteins; LM, lipid metabolism; IMR, intermediary metabolism and respiration; ISP, insertion sequences and phages; IP, information pathways; CH, conserved hypotheticals; CWP, cell wall and cell processes. (B) Heat map depicting genes involved in lipid metabolism that are differentially expressed in Mtb^A^ relative to Mtb^P^. (C) Thin-layer chromatogram of bacteria isolated from 3T3L1 adipocytes labeled with [^14^C]oleate (lane 1) or [^14^C]acetate (lane 2) or from M. tuberculosis grown *in vitro* (lane 4). Lane 3 contains a triglyceride (TG) standard. (D) Thin-layer chromatogram of lipids resolved in petroleum ether–diethyl ether (9:1). Lipids were from Mtb^A^, Mtb^P^, and Mtb^C^ at 10 days postinfection or postinoculation. S, triglyceride standard. (E) Densitometric quantification of triglyceride as a percentage of total lipids. Data are means ± standard errors of the means for three independent experiments. **, *P* < 0.01. (F) Luminescence of H37Rv expressing *luxCDABE* in adipocytes and preadipocytes. Data are means ± standard deviations for three wells and represent three independent experiments. RLU, relative light units.

When we classified differentially expressed genes based on TubercuList classification, we found an expansion of the cluster of genes involved in lipid metabolism (Fig. 3A and B). M. tuberculosis encodes two types of fatty acid synthases: type I, a multifunctional polypeptide encoded by *fas*, and type II, a multienzyme complex involved in the synthesis of fatty acids with a carbon chain length longer than C_26_ (15). Fatty acid biosynthesis by both systems is initiated by the condensation of malonyl coenzyme A (malonyl-CoA) to an acyl carrier protein, encoded by the *acpA* gene. Mtb^A^ exhibited 2-fold downregulation of *acpA* and 2.4-fold downregulation of *fas* (Fig. 3B; also File S1 in the supplemental material). *fabG4* and *kasB*, components of the type II system, were also downregulated (Fig. 3B). The downregulation of *fas* was further validated by quantitative real-time PCR (qRT-PCR) (see Fig. S2 in the supplemental material). The decreased expression of these genes in Mtb^A^ suggested that M. tuberculosis was sustained by adipocyte-derived fatty acids rather than expending resources on *de novo* synthesis of fatty acids.

To check if host-derived fatty acids could be utilized for bacterial lipid synthesis, we first labeled adipocytes with [^14^C]acetate or [^14^C]oleate and then infected them with H37Rv. After growth over a 10-day period, we isolated lipids from bacilli and performed thin-layer chromatography (TLC) to assess the incorporation of radioactivity into the bacillary fraction. The TLC indicated that a single lipid species was radiolabeled (Fig. 3C). These data revealed that bacilli growing on necrotic adipocytes utilized host-derived metabolites in their triglyceride fractions. In contrast, no radioactivity was detectable in phthiocerol dimycocerosate (PDIM). Similarly, [^14^C]acetate could also be traced into the polar lipid pool of Mtb^A^ (data not shown). This suggested preferential utilization of host-derived metabolites for glycerolipids.

Next, we asked if Mtb^A^ had a higher total triglyceride content than Mtb^P^. After cells were infected at a multiplicity of infection (MOI) of 1 for 24 h, bacilli were recovered at 10 days postinfection. We observed that over a 10-day period of infection, growth on necrotic adipocytes led to triglyceride levels 6-fold higher than those in bacteria grown on necrotic preadipocytes or in culture medium (Fig. 3D and E).

A recent study reported differential expression of lipases and triglyceride synthases in M. tuberculosis at day 6 after adipocyte infection (16). In the present study, we found no significant changes in the expression levels of the putative lipases at day 10. Of the 15 genes encoding putative triglyceride synthases (17), only Rv3371 and *tgs4* were upregulated, whereas *tgs1* was downregulated, in Mtb^A^ (Fig. 3B; see also Fig. S3A in the supplemental material), hinting at specific regulation of possibly redundant genes for triglyceride synthesis. By qRT-PCR, we confirmed that Rv3371 was 3-fold upregulated in Mtb^A^ (Fig. S3B). Interestingly, by qRT-PCR, we found that although *tgs1* was tending toward downregulation at day 10, it was induced at day 5, while the reverse trend was observed for Rv3371 (data not shown), suggesting temporal regulation of the triglyceride synthesis machinery. *tgs1* is a dormancy-related gene, regulated by the transcription factor DevR (18, 19); downregulation of *tgs1* was suggestive of downregulation of the *devR* regulon. Indeed, qRT-PCR-based analysis confirmed that, like *tgs1* expression, the expression of *narX*, *narK2*, and *devR* was decreased in Mtb^A^ from that in Mtb^P^ (Fig. S3C).

Genes involved in complex lipid metabolism showed differential behavior. Among the genes encoding polyketide synthetases, *pks6* was downregulated, whereas *pks8* and *pks2* were upregulated, in Mtb^A^. Surprisingly, genes involved in the synthesis of the complex lipid siderophore mycobactin were downregulated in Mtb^A^ relative to Mtb^P^, indicating differential iron availability in the two necrotic cellular systems.

### Metabolic activity of M. tuberculosis in adipocytes.

We also found that a few genes involved in β-oxidation were differentially expressed in Mtb^A^ relative to Mtb^P^ at day 10 postinfection (Fig. 3B). Fatty acid oxidation requires FadD, FadA, FadB, and the Ech proteins, all of which have multiple functional homologues encoded by the M. tuberculosis genome. We found induction of *fadA6* (2-fold), *fadA4* (1.75-fold), and *fadE5* (1.68-fold) in Mtb^A^ relative to Mtb^P^. The induction of *fadA6* in Mtb^A^ suggests that this gene may be required for the utilization of host-derived longer-chain fatty acids, since it is not required for the utilization of C_12_ fatty acid (20). The upregulation of *fadE5* and *pks2* suggests that bacilli encounter a cholesterol-rich environment in necrotic adipocytes (21). A peripheral membrane fatty acyl-CoA synthase, FadD13, thought to be involved in the degradation of long-chain fatty acids (22), also showed 1.7-fold-higher expression in Mtb^A^ than in Mtb^P^.

Interestingly, Mtb^A^ also exhibited downregulation of *fadB3*, encoding an enzyme required for the penultimate step in fatty acid oxidation (23). The specific downregulation of *fadB3* and upregulation of *fadA6* in Mtb^A^ suggest that transcriptional regulation of the fatty acid oxidation machinery may be important for optimal utilization of available fatty acids within the host.

To test if Mtb^A^ was also metabolically more active than Mtb^P^, we utilized the autoluminescence system of Photorhabdus luminescens, which reports on metabolic activity using cellular fatty acid intermediates (24, 25). Reduced flavin mononucleotide (FMNH_2_), NADPH, and ATP are required for light production, and therefore, the assay reports on total metabolic activity while the substrate is a cellular fatty acid. We measured the luminescence of H37Rv expressing the *luxCDABE* operon (H37Rv/GC-Lux) during the infection of adipocytes and preadipocytes. Bacterial luminescence in adipocytes was significantly higher than that in preadipocytes at all time points (Fig. 3F), irrespective of similar bacterial numbers (Fig. 1E). This showed that mycobacterial metabolic activity using fatty acids as the substrate is indeed higher in adipocytes than in preadipocytes at time points when the bacteria are intracellular and extracellular. Together, these data revealed that Mtb^A^ is in a metabolically active state, with the ability to oxidize fatty acids and simultaneously store excess fatty acids as triglycerides.

### M. tuberculosis encounters differential iron availability in adipocytes.

**FIG 4:**
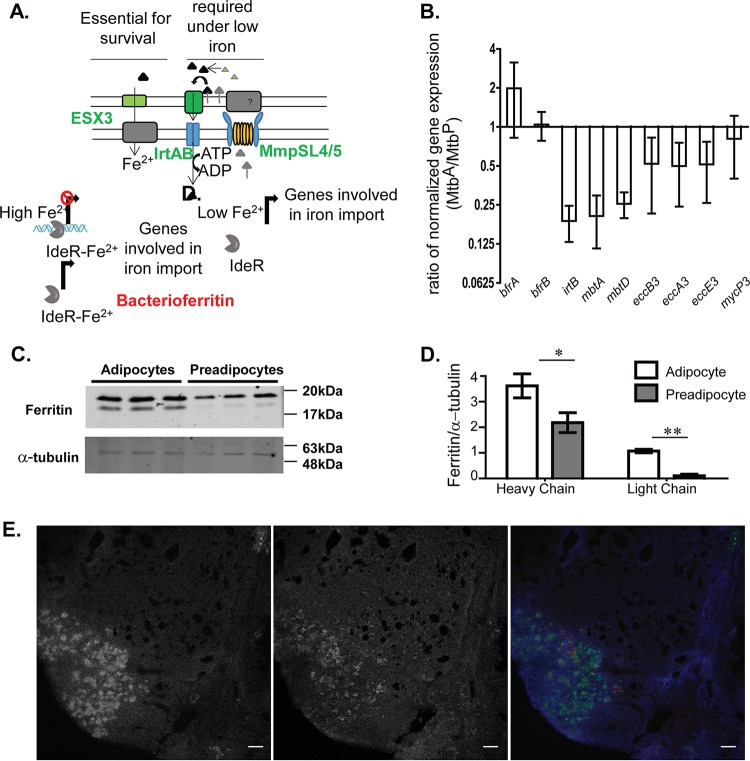
Iron homeostasis of M. tuberculosis in adipocytes relative to that in preadipocytes. (A) Schematic representation of the iron homeostasis pathway in M. tuberculosis. (B) qRT-PCR-based validation of iron-regulated genes of M. tuberculosis showing fold changes in the normalized gene expression of bacilli growing in adipocytes from that of bacilli in preadipocytes 10 days postinfection. Data are means ± standard errors of the means for three independent experiments. (C) Immunoblotting of adipocytes and preadipocytes with anti-ferritin and anti-α tubulin. (D) Quantitation of ferritin and α-tubulin in adipocytes and preadipocytes. Data are means ± standard deviations for three wells. *, *P* < 0.05; **, *P* < 0.01. (E) Differential ferritin levels in lipid-rich regions of the mouse granuloma. (Left) Bodipy 493/503 staining; (center) immunofluorescence signal for ferritin; (right) pseudocolored merge where blue indicates DAPI staining, green indicates Bodipy 493/503 staining, and red indicates ferritin immunofluorescence. Bars, 100 μm.

Differentially expressed genes belonging to the lipid metabolism class included genes involved in mycobactin synthesis (Fig. 3B). A parallel transcription factor network analysis of the differentially expressed genes revealed that the entire IdeR regulon was downregulated in Mtb^A^ relative to Mtb^P^ (see Fig. S4 and S5 in the supplemental material). IdeR is an iron-responsive repressor which, in the Fe^2+^-bound state, binds as a dimer to its target DNA (26, 27). Decreased availability of Fe^2+^ results in the removal of IdeR from its target DNA, thereby derepressing target gene expression. This results in enhanced expression of genes involved in scavenging iron from the extracellular medium using carboxymycobactin secretion (*mbtK-mbtN*, *mbtA-mbtJ*, and *mmpL4*) and iron uptake (*irtA*, *irtB*, and Esx3 component genes) (27, 28). Suppression of *mbtK-mbtN* (2-fold), *mbtA-mbtJ* (2- to 3-fold), *irtA* (3-fold), *irtB* (4-fold), and *mmpL4* (3-fold) in Mtb^A^ suggested higher iron availability for M. tuberculosis in necrotic adipocytes than in necrotic preadipocytes (Fig. S5). Even in comparison to growth in DMEM, Mtb^A^ exhibited repression of the IdeR regulon, suggesting a repression unique to the availability of iron from adipocytes (File S1 in the supplemental material). The type VII secretion system Esx-3 is also involved in iron and zinc uptake in M. tuberculosis under iron-limiting as well as iron-sufficient conditions and relies on mycobactin-dependent uptake of iron (29). The expression of genes of the Esx-3 locus, Rv0282 to Rv0292, was 2-fold lower in Mtb^A^ than in Mtb^P^ (Fig. S5). By qRT-PCR, we confirmed that *mbtA*, *mbtD*, *irtB*, and two of the *esx-3* genes were differentially expressed in Mtb^A^ relative to Mtb^P^ (Fig. 4B). These data suggested that M. tuberculosis limits the uptake of iron from the host more when grown on adipocytes than when grown on preadipocytes.

To understand if indeed the adipocyte environment contained higher levels of iron than the preadipocyte environment, we quantified iron using inductively coupled plasma mass spectrometry (ICP-MS). This analysis indicated approximately 1.3-fold-higher total-iron levels in adipocytes than in preadipocytes (25.5 ± 5.2 versus 13.1 ± 4 pg/liter, respectively) (*P* = 0.06). We checked ferritin levels as a measure of differential iron metabolism in the two host cell types. Levels of ferritin heavy and light chains were found to be 1.8- and 2.3-fold higher on adipocytes than on preadipocytes (Fig. 4C and D). Mycobacterial siderophores can scavenge iron from mammalian ferritin (30). Increased availability of host ferritin therefore limits the requirement for siderophore production and export by mycobacteria.

We further compared ferritin expression in RAW 264.7 macrophages with and without oleic acid treatment; expression of the light chain of ferritin was found to be increased after oleic acid treatment for 48 h (see Fig. S6 in the supplemental material). This further demonstrated that the relationship between neutral lipid content and ferritin expression is widespread across cell types. We also sought to investigate if ferritin availability is associated with lipid availability within tuberculosis (TB) granulomas. We infected C3HeB/FeJ mice with H37Rv at 100 CFU/lung and investigated granulomas at day 49 postinfection for the expression of ferritin. Sections that were probed with anti-ferritin antibodies by immunofluorescence were stained with Bodipy 493/503 to detect foamy macrophages. Sections were counterstained with DAPI. Granulomatous regions exhibited enrichment of Bodipy 493/503 to specific cells within granulomas (Fig. 4E, left). Ferritin immunostaining was found to be enriched in the Bodipy 493/503-positive regions of the granulomas (Fig. 4E, center and right). These data confirmed that even in the context of a TB granuloma, lipid-rich cellular niches are present with increased ferritin and possibly increased iron availability for M. tuberculosis.

### M. tuberculosis mitigates oxidative stress in the necrotic adipocyte model.

**FIG 5:**
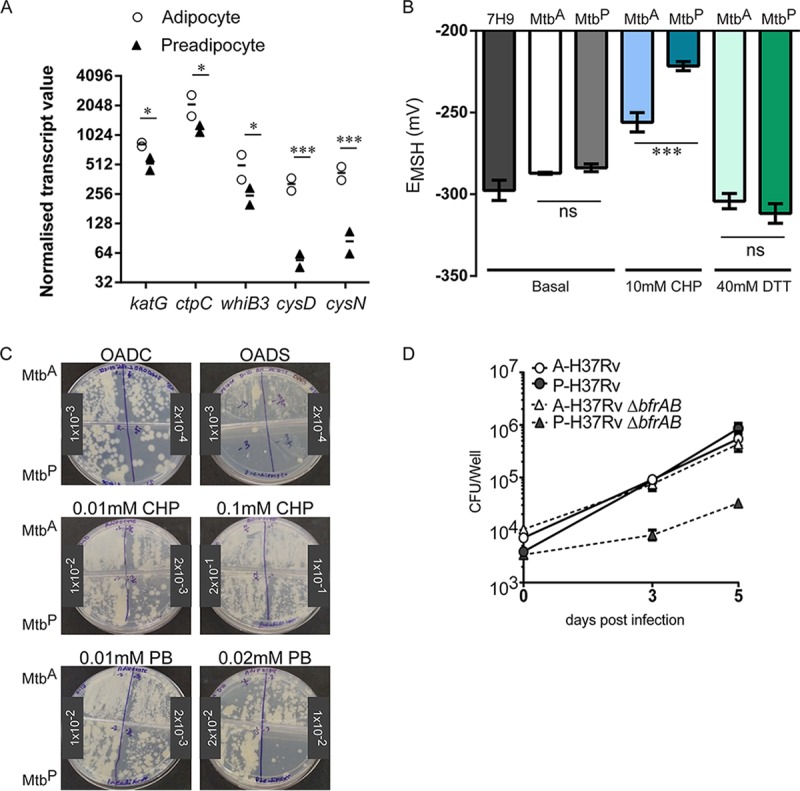
Differential response of Mtb^A^ and Mtb^P^ to oxidative stress. (A) Normalized transcript abundances of differentially expressed genes involved in redox stress mitigation in Mtb^A^ and Mtb^P^ 10 days postinfection. (B) Cytosolic redox potential (E_MSH_) under basal conditions and in response to 10 mM cumene hydroperoxide (CHP) or 40 mM dithiotreitol (DTT) in Mtb^A^ and Mtb^P^ 10 days postinfection. *, *P* < 0.05; ***, *P* < 0.001. (C) Growth assay of Mtb^A^ and Mtb^P^ 10 days postinfection in the presence of different concentrations of oxidizing agents (0.1 and 0.01 mM CHP and 0.01 and 0.02 mM plumbagin [PB]) in 7H10 OADC or OADS plates. (D) Growth kinetics of Δ*bfrAB* and wild-type H37Rv in adipocytes (A) and preadipocytes (P) as assessed by CFU counts. Data are means ± standard deviations for three wells (representative of three experiments).

Apart from being a micronutrient, iron in the ferrous form is a major contributor to oxidative stress by participating in the Fenton reaction. To understand if Mtb^A^ was facing oxidative stress, we looked for the expression of genes involved in the response to oxidative stress from the list of differentially expressed genes (*P* < 0.05). Higher expression of *katG*, *ctpC*, *whiB3*, *cysD*, and *cysN* indicated that Mtb^A^ did indeed exhibit an active oxidative-stress response (Fig. 5A). Oxidative stress leads to dismantling of the iron-sulfur cluster in IdeR, thereby leading to the repression of iron storage genes, such as *bfrB*, and the induction of iron acquisition genes of the *mbt* clusters, presumably to rebuild the Fe-S cluster (31). In addition, the cysteine biosynthesis genes CysM and CysN are induced to build the Fe-S cluster. Lower expression of IdeR-repressed genes in the face of an active antioxidant response suggested that possibly Mtb^A^ is able to mitigate oxidative stress in the adipocyte environment. To further understand if M. tuberculosis mitigates oxidative stress in the necrotic adipocyte environment, we made use of the mycothiol (MSH)-sensitive green fluorescent protein (GFP) as a surrogate for cytosolic redox potential (E_MSH_) (32). The basal E_MSH_ values of Mtb^A^ and Mtb^P^ were indistinguishable and similar to that of M. tuberculosis grown in 7H9 medium. Addition of the oxidizing agent cumene hydroperoxide (CHP) at 10 mM led to oxidation of the cytosol of Mtb^P^, with an increase in E_MSH_ from −283.8 ± 2.3 mV to −221.6 ± 2.8 mV (*P* < 0.0001). However, 10 mM CHP could oxidize the cytosol of Mtb^A^ from −287.2 ± 0.6 to only −255.9 ± 5.92 (*P* < 0.01). In contrast, 40 mM dithiothreitol (DTT) was able to reduce the cytosol of Mtb^A^ and Mtb^P^ to similar extents. The apparent resistance to oxidation suggested that M. tuberculosis acquired an oxidative-stress mitigation capacity in the adipocyte model of infection. To test if the cytosolic redox potential correlated with increased resistance to oxidative stress, dilutions of Mtb^A^ and Mtb^P^ at day 10 of infection were plated onto 7H10 plates containing different concentrations of the oxidative-stress-inducing agents plumbagin (PB) and CHP. To maximize toxicity due to the oxidants, these agents were added to plates supplemented with oleic acid-albumin-dextrose-saline (OADS) rather than oleic acid-albumin-dextrose-catalase (OADC), since the presence of catalase in the latter mixture protected against oxidative-stress-mediated killing. Interestingly, replacing OADC with OADS led to reduced viability for Mtb^P^ but had no effect on the viability of Mtb^A^ (Fig. 5C). Similarly, CHP at 0.01 mM and 0.1 mM, and PB at 0.01 mM and 0.02 mM, affected the viability of Mtb^P^ to a greater extent than that of Mtb^A^ (Fig. 5C), in support of our hypothesis that Mtb^A^ is better able to mitigate oxidative stress than Mtb^P^.

Iron storage in M. tuberculosis is linked to its ability to counter oxidative stress (33, 34). A bacterioferritin double deletion mutant (Δ*bfrAB*) is attenuated for growth under conditions of excess iron and oxidative stress but not in normal growth media (33, 35). We hypothesized that mycobacteria that are sensitive to iron-mediated oxidative stress would therefore be protected in a host environment that favors the mitigation of oxidative stress by M. tuberculosis. To test this hypothesis, we compared the growth of a genetic deletion mutant of *bfrAB* in adipocytes and preadipocytes (35). The *bfrAB* deletion mutant of M. tuberculosis H37Rv was attenuated for growth in preadipocytes (Fig. 5D). In contrast, this mutant exhibited no growth impairment in adipocytes (Fig. 5D), further supporting the hypothesis that the adipocyte environment provides protection from iron-mediated oxidative stress to M. tuberculosis.

### Fatty acids and ferritin can rescue the growth of the *bfrAB* deletion mutant of M. tuberculosis.

**FIG 6:**
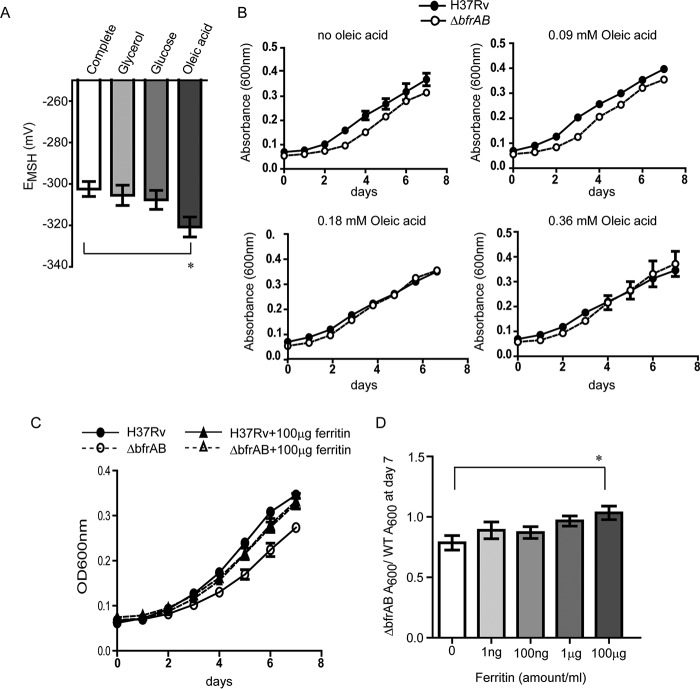
Rescue of *in vitro* growth of the Δ*bfrAB* mutant by oleic acid and ferritin. (A) Cytosolic redox potential (E_MSH_) of M. tuberculosis after growth in different carbon sources (complete medium) or in a medium containing a single carbon source (glycerol, glucose, or oleic acid). (B) Absorbance at 600 nm of wild-type and Δ*bfrAB* M. tuberculosis grown in a glycerol- and glucose-containing medium in the absence or presence of oleic acid at the indicated concentrations (representative of three experiments). (C) Absorbance at 600 nm of wild-type and Δ*bfrAB* M. tuberculosis in a glycerol-containing medium in the absence or presence of 100 μg/ml ferritin for 7 days. (D) Ratio of the absorbance at 600 nm of M. tuberculosis Δ*bfrAB* to that of the wild type (WT) 7 days after growth in a glycerol medium in the absence or presence of different concentrations of ferritin (given below the graph) from three independent experiments. *, *P* < 0.05.

To understand whether and how carbon sources from the host and the elevated ferritin levels in adipocytes may contribute to the increased oxidative-stress mitigation capacity of M. tuberculosis, we studied the effects of these factors on the E_MSH_ of M. tuberculosis under defined medium conditions. Fatty acids are one of the major carbon sources that Mtb^A^ encounters. To rule out the contribution of Tween 80 as a carbon source, we added 0.025% tyloxapol in these experiments. The complete medium contained 0.18 mM oleic acid, 0.5% glycerol, and 0.2% dextrose, equivalent to the 7H9 complete medium used earlier. E_MSH_ was found to be higher when glycerol or glucose was used as the sole carbon source than when oleic acid was the sole carbon source (Fig. 6A). This demonstrated that the metabolism of oleic acid leads to a more-reducing cytosol. Next, we asked if the addition of oleic acid to a medium containing glycerol and glucose alters the E_MSH_ of the bacterial cytosol. We found that the addition of increasing concentrations of oleic acid to a medium containing glycerol and glucose showed a trend toward reducing the E_MSH_ of bacterial cytosol (see Fig. S7 in the supplemental material).

To understand if the lowering of the cytosolic redox potential can rescue the growth of the otherwise oxidative-stress-sensitive mutant, we compared the growth of the mutant and its wild-type parent strain in the presence of different carbon sources. The growth of the Δ*bfrAB* mutant was attenuated in a medium containing glycerol and glucose and was rescued by the addition of oleic acid to a concentration of 0.18 mM or 0.36 mM (Fig. 6B).

We asked if the addition of ferritin can also rescue the growth defect of the Δ*bfrAB* mutant in a glycerol medium. We found that the addition of 100 μg/ml ferritin was able to rescue the growth of the Δ*bfrAB* mutant, and the mutant also showed better growth with increasing concentrations of ferritin (Fig. 6C and D). However, the addition of ferritin to a glycerol medium was not able to alter the cytosolic redox potential in wild-type M. tuberculosis (data not shown). This rescue phenomenon was likely due to increased availability of iron, since the addition of apoferritin was not able to rescue the growth of the mutant (see Fig. S8 in the supplemental material).

Together, these data demonstrate that as M. tuberculosis undergoes oxidative metabolism of fatty acids, it shifts to reducing its cytosol, allowing it to overcome oxidative stress. The presence of ferritin, which is a host homeostatic response to increased fatty acids, further provides requisite amounts of iron to the bacteria. M. tuberculosis grown in a fatty-acid-containing medium, in contrast to a dextrose-containing medium, does not exhibit differential expression of iron metabolism genes (8) (see File S3 in the supplemental material), suggesting that synergy between fatty acids and iron availability is linked to the lipid-rich host environment.

## DISCUSSION

In this study, we utilized the adipocyte model of M. tuberculosis infection to mimic bacterial physiology in a necrotic lipid-rich host environment. Our study highlights the end-stage, extracellular state in which mycobacteria reside in a lipid-rich environment, akin to the caseous environment encountered by M. tuberculosis in a necrotic granuloma (7, 36). We described differential expression of protein-coding genes and, on the basis of transcription factor network analysis, identified iron response as a major signature of these bacteria. We show that the host environment, besides being a rich source of fatty acids, is also enriched in ferritin, an iron carrier. We demonstrate that oleic acid, a host-derived fatty acid, induces resistance to oxidation of the mycobacterial cytosol. This resilience helps an otherwise iron-mediated oxidative-stress-sensitive mutant of M. tuberculosis (Δ*bfrAB*) to grow in the adipocyte model, while it remains attenuated in preadipocytes. Additionally, host ferritin likely provides an iron-rich environment, which is limiting in preadipocytes.

Previous work that has addressed the physiology of bacteria under *in vitro* conditions of fatty acid availability showed differential expression of genes involved in lipid metabolism and redox mitigation (8), findings similar to those in our cellular model (File S3 in the supplemental material). In this work, we delineate a functional coupling of ferritin and increased fatty acid availability in the context of cellular infection. This aspect seems to be unique to the host cell physiology. High ferritin levels are also associated with foamy macrophages in atherosclerosis (37) and during fibroblast-to-adipocyte differentiation (38), where ferritin is proposed to protect cells from oxidative stress. The ability of host ferritin to load and offload iron might provide the requisite buffering of free iron, which, in an otherwise oxidative environment, may be damaging to the bacteria. Specific distribution of high ferritin expression in lipid-rich macrophages of infected C3HeB/FeJ mice further validates the lipid-iron link we established using the adipocyte model of infection. Earlier work has also shown that caseous granulomas from human tuberculosis patients are associated with higher expression of ferritin (36). In addition, the guinea pig model of infection provided evidence for increased ferritin levels in cells peripheral to the necrotic core and increased extracellular iron within the acellular core of the TB granuloma (39). Increased availability of host ferritin therefore limits the requirement for siderophore production and export by mycobacteria.

BfrA has been shown to be dispensable for iron-dependent growth of M. tuberculosis, while BfrB is required for optimal growth in an iron-dependent manner (34). The role of BfrB is particularly complex: not only does it provide protection against high iron levels, but it also enables growth under conditions of iron limitation (34). However, these genes synergize in the protection of M. tuberculosis against oxidative stress (33). Attenuation of the double mutant in guinea pigs could be due either to direct iron-dependent killing of the mutant or to sensitivity to oxidative stress (35). Our data show that in lipid-rich cells, the *bfrAB* mutant could be protected. Our data indicate that ferritin and fatty acids, both of which are more abundant in adipocytes than in preadipocytes, offer protection to this mutant in otherwise growth-limiting broth culture. Our data suggest that protection from oxidation of the cytosol may be responsible for the benefit offered by oleic acid. Previous work with *bfrA*, *bfrB*, and *bfrAB* double deletion mutants has revealed that both *bfrB* and *bfrAB* mutants are attenuated in mouse and guinea pig models of infection. We predict that the same phenotype would be found in C3HeB/FeJ mice; lipid-rich foamy macrophages would appear during the course of infection, prior to which the *bfrAB* mutant would already be attenuated. As these new niches develop, *bfrAB* might become nonessential for mycobacterial growth, while these genes would continue to be required for bacterial growth in nonfoamy macrophages.

The adipocyte model was first described by Neyrolles et al. as a model for mycobacterial dormancy (40), while we describe a scenario of higher metabolic activity of M. tuberculosis using adipocytes as the host. One of the key differences between that study and ours is the finding that adipocytes are able to sustain the growth of M. tuberculosis H37Rv, while the clinical strain used in the earlier study underwent nonreplicating persistence. We suspect that this could be due to inherent strain differences, since subsequent studies that used H37Rv also found growth of M. tuberculosis in adipocytes (41, 42). Our model is a chronic infection model such that we capture bacilli attached to the necrotic cells on which they have grown. Recently, there has been interest in trying to understand mycobacterial physiology in environments that mimic the extracellular stage of infection in necrotic and cavitary lesions (7, 43). Attachment of bacteria to host components as a complex biofilm-like structure contributes to the formation of drug-tolerant extracellular bacilli (43). Our data describe the formation of extracellular cords of bacteria attached to the necrotic host cell components. Due to the complex nature of the infection model, where bacteria grew as cords attached to necrotic cells, an accurate assessment of individual bacillary counts that were intracellular or extracellular could not be made. At least 90% of the cords were definitely extracellular in both cellular models at the time of transcriptome profiling. Intracellular bacteria might be facing hypoxia and iron limitation, while extracellular bacteria would be sufficiently oxygenated and would encounter adequate iron. Had we sampled the bacteria at earlier time points, lower expression of hypoxia and iron response genes in the adipocyte infection could be due to slightly higher numbers of extracellular bacilli in the adipocyte model than in the preadipocyte model. The relatively lower expression of iron-scavenging genes in adipocyte-grown bacteria is likely not due to this difference at day 10, since even compared to medium-grown bacilli, adipocyte-grown bacilli had lower abundances of iron-scavenging gene transcripts. The current model should be further improved to incorporate features of hypoxia that bacilli are likely to encounter within a granuloma in the necrotic core (44). Our findings may have wider implications for the control of bacteria in caseous granulomas and also for other human pathogens that encounter lipid-rich environments, such as Staphylococcus aureus and Trypanosoma cruzi (45, 46). Further genetic approaches are required to understand pathways that are essential for the survival of bacteria in this environment. In addition, oxidative stress is inherent to the mechanism of action of isoniazid, a frontline TB drug (32). Adipocyte-grown M. tuberculosis exhibits tolerance to isoniazid (40; also data not shown). Therefore, inhibiting pathways that are essential for oxidative stress mitigation under lipid-rich conditions might increase the efficacy of this antibiotic.

## MATERIALS AND METHODS

### Bacteria and mammalian cell culture.

M. tuberculosis strains were grown in Middlebrook 7H9 medium containing albumin-dextrose-catalase (ADC; BD Difco) at 37°C under shaking conditions unless stated otherwise. The *bfrAB* deletion mutant and its wild-type control strain (H37Rv) were a kind gift from Anil Tyagi (35). Stable mCherry expression in H37Rv was attained using pCherry3 (plasmid 24659; Addgene), a kind gift from Tanya Parish. The pMrx1-roGFP2 plasmid, a kind gift from Amit Singh, was electroporated in H37Rv to obtain Mrx1-roGFP2 expressing H37Rv. 3T3L1 fibroblasts were a kind gift from David Savage (source, European Collection of Authenticated Cell Cultures). The 3T3L1 fibroblasts were cultured in Dulbecco's modified Eagle medium (DMEM; Invitrogen) containing 10% heat-inactivated neonatal calf serum (NCS; Sigma) at 37°C under 5% CO_2_. Differentiation of preadipocytes was initiated 2 days postconfluence by adding 250 μM 3-isobutyl-1-methylxanthine (IBMX), 1 μM dexamethasone, and 10 μg/ml human insulin to DMEM containing 10% heat-inactivated fetal bovine serum (FBS). On the third day, the medium was replaced with DMEM–10% FBS containing insulin only. The medium was changed to add DMEM–10% FBS (complete DMEM) at day 5 and day 8. Cells were infected such that adipocytes had been under differentiation for 10 days and preadipocytes had been confluent for 2 days.

Human subcutaneous and visceral preadipocyte cells were purchased from Lonza (catalog no. PT-5001 and PT-5005). They were cultured in PGM-2 basal medium (catalog no. PT-8202) at 37°C under 5% CO_2_. Differentiation of preadipocytes was initiated 2 days postconfluence by adding necessary supplements provided in the PGM-2 SingleQuot kit (catalog no. PT-9502). Cells were kept in this medium for 10 days for complete differentiation.

RAW 264.7 cells (source, European Collection of Authenticated Cell Cultures) were grown in RPMI medium containing 10% heat-inactivated FBS at 37°C under 5% CO_2_.

All cell lines were routinely checked for mycoplasma using MycoAlert assay reagents (Lonza) and were verified to be mycoplasma free.

### Mycobacterial infection and survival assay.

Mature adipocytes and preadipocytes were infected at a multiplicity of infection (MOI) of 1 bacterium per cell for 4 or 24 h at 37°C under 5% CO_2_. With human adipocytes, infections were performed at an MOI of 1 for 24 h, since bacterial uptake was slower in these cells. At this point, infection was stopped by removing the medium and washing four times in phosphate-buffered saline (PBS). Cells were further incubated in fresh DMEM containing 10% FBS. At various time points, cells were lysed in distilled water containing 0.1% Triton X-100 (Sigma) for preadipocytes and 1% Triton X-100 for adipocytes, and lysates were plated at various dilutions on 7H10 agar supplemented with oleic acid-albumin-dextrose-catalase (OADC). These concentrations of detergents were optimized to recover the maximum number of bacteria from each condition (ensuring the disruption of cords) without causing detergent-induced toxicity.

### Assessment of cell death.

Differentiated and undifferentiated adipocytes were infected with mCherry expressing H37Rv at an MOI of 1 for 24 h. At each time point postinfection, cells were stained with Sytox green (catalog no. S34860; Molecular Probes) in PBS for 30 min, washed in PBS, and then fixed with 4% formaldehyde in 1× PBS. Fixed cells were stained with DAPI. DAPI-positive and Sytox green-positive cells were counted manually after taking images of five representative fields of each of the triplicate samples for each time point. Sytox green positivity was calculated as the percentage of DAPI-stained nuclei stained with Sytox green.

### Quantitation of extracellular and intracellular bacterial populations.

Differentiated adipocytes and undifferentiated preadipocytes were infected with mCherry expressing H37Rv at an MOI of 1 for 24 h. At each time point postinfection, cells were fixed with 4% formaldehyde in 1× PBS. Fixed cells were used for immunostaining without permeabilization. Briefly, nonspecific staining was blocked with 3% bovine serum albumin (BSA) in PBS for 1 h at room temperature, and cells were then incubated with an anti-M. tuberculosis antibody (anti-lipoarabinomannan [anti-LAM]; Meridian Life Science) in 1% BSA in PBS overnight at 4°C. Incubation with an Alexa Fluor 633-conjugated anti-rabbit secondary antibody (catalog no. 913915; Invitrogen) was also carried out in 1% BSA in PBS for 1 h at room temperature. The cells were then stained with DAPI, and imaging was done using a confocal microscope. Five z-stacks were acquired per experiment, and averages from three independent experiments were computed. Uninfected cells were used as negative controls, and background fluorescence for the far-red channel was used as a threshold to qualify the anti-LAM signal as a true signal. Single bacilli were countable at 24 h but not with confidence at days 5 and 10, so cords rather than single bacteria were counted at these two time points. Those staining above threshold were counted as extracellular, and those below threshold were counted as intracellular.

### RNA isolation and RNA sequencing.

Infected adipocytes, infected preadipocytes, and DMEM-grown bacilli were harvested at the 10th day after infection at an MOI of 1 for 24 h by adding 4 M guanidine isothiocyanate (GITC) to the flasks in an amount equal to that of the medium and scraping cells. The suspension was spun down at 3,500 rpm for 5 min, and the pellet was resuspended in 1 ml TRIzol LS reagent (Ambion), followed by bead beating for RNA isolation by standard methods. Column-purified RNA was DNase treated using Turbo DNase (catalog no. AM2238; Ambion) for 1 h at 37°C. RNA was qualitatively and quantitatively estimated using an Agilent 2100 bioanalyzer. Subsequently, rRNA was depleted using a Ribo-Zero Magnetic Gold kit (Epidemiology) (Epicentre), followed by sequencing library preparation using a ScriptSeq v2 RNA-seq library preparation kit (Epicentre). Samples were sequenced on an Illumina MiSeq platform using 150-cycle paired-end chemistry with MiSeq v3 kits. RNA from two independent experiments, each with adipocyte-, preadipocyte-, and DMEM-grown bacilli, was sequenced in the same manner, and data from both experiments were used for analysis.

### Transcriptome profiling and differential expression analysis.

Sequencing reads from MiSeq were checked for quality using FastQC and were aligned to the Mycobacterium tuberculosis H37Rv reference genome (NCBI accession no. NC_000962) with Bowtie 2 (47) in “–very-sensitive” mode. We performed postalignment processing with SAMtools (48) to put the alignment data into a format suitable for downstream analysis. To quantitate the expression levels of genes, we generated a gene read count matrix using TubercuList annotations (49) with htseq-count (50). htseq-count was run on “intersection-strict” with a minimum alignment quality of 10. Statistical tests for differential expression were performed with the DESeq2 package (51) of the Bioconductor suite in R. We used the pipeline command of DESeq2 to read the count matrix and perform subsequent normalization and differential expression analysis. For final results, we disabled the default Cook's distance-based filtering of outlier genes and qualified genes as significantly differentially expressed only if they had a *P* value of <0.05.

### Transcriptional factor–target interaction network.

We generated a transcription factor–target gene regulatory interaction network for all the differentially expressed genes we obtained from our transcriptome analysis. Regulatory interactions were obtained from the recently described M. tuberculosis regulatory network resource, as well from manual literature curation for interactions corresponding to fatty acid metabolism and iron metabolism (28, 52–55). The type of regulatory interaction was documented if available. We summarized the differentially expressed genes and their regulatory interactions in a Circos image, where genes have been grouped into three categories for transcription factors, significantly overexpressed genes, and significantly underexpressed genes.

### qRT-PCR.

RNA was extracted in three independent experiments as described above. cDNA, prepared using Moloney murine leukemia virus (M-MuLV) reverse transcriptase (ProtoScript II), was utilized for quantitative real-time PCR (qRT-PCR) with SYBR green in a Roche LightCycler 480 instrument II. Absolute quantitation was performed using genomic DNA from M. tuberculosis. Template normalization was performed by dividing the absolute gene expression of specific genes by the absolute gene expression of 16S rRNA. The sequences of primers used for qRT-PCR are provided in File S4 in the supplemental material.

### Extraction and analysis of lipid.

Ten million adipocytes and preadipocytes were infected at an MOI of 1 for 24 h. Infected adipocytes and preadipocytes, along with DMEM-grown bacilli, were harvested on the 10th day after infection by scraping out the cells in the medium and pelleting down at 3,500 rpm for 5 min. Apolar and polar lipids were extracted after host cell lysis. Host cells were lysed by adding deionized water to the pellet. To remove host-derived lipid, this pellet was first washed twice in deionized water and then twice in 0.1% Triton X-100, and finally, the bacterial pellet was collected. The bacterial pellet was then inactivated by heating at 95°C for 15 min. Uninfected adipocytes and preadipocytes were also lysed by following the same protocol to check for host-derived lipid contamination. The dry weight of the sample was used for normalization of the lipid extract. Apolar lipids were extracted by adding 2 ml of a methanolic solution of 0.3% sodium chloride and 1 ml of petroleum ether (60 to 80°C) to the dried cell pellet. The cell suspension was mixed in a tube rotator for 30 min, followed by centrifugation at 2,000 rpm for 2 min. The upper layer, containing apolar lipids, was collected in a separate vial, and 1 ml of petroleum ether was added to the lower layer, which was then mixed for 15 min. The extract was again centrifuged to collect the upper layer. The pooled apolar lipids were dried at 50°C.

To make a total-lipid extract from 3T3L1 adipocytes or preadipocytes, the method of Bligh and Dyer (56) was followed. Briefly, the cells were first washed twice with PBS and then lysed in 1% Triton X-100. After lysis, 4 volumes of methanol-chloroform (2:1) were added, and the lysate was vortexed. One volume of 50 mM citric acid, 1 volume of water, and 1 volume of chloroform were added, and the mixture was vortexed. It was then centrifuged at 10,000 rpm for 10 min, after which the lower phase was taken and dried.

### Lipid analysis by thin-layer chromatography.

Equivalent amounts of apolar (normalized to biomass) lipids or a dried lipid extract from mammalian cells (from an equal number of cells) suspended in chloroform-methanol (2:1, vol/vol) were then spotted onto TLC plates and were analyzed for triglyceride by using a solvent system of petroleum ether–diethyl ether (9:1) at room temperature or by using hexane–diethyl ether–acetic acid (70:30:1) at 4°C. For the visualization of unlabeled lipids on TLCs, the TLCs were stained using 10% (wt/vol) copper sulfate in an 8% (vol/vol) phosphoric acid solution, followed by charring at 150°C.

For densitometric analysis of bacterial triglyceride after subtraction of the densitometric values of the triglyceride fraction obtained by the lysis of uninfected cells from the respective bacterial samples, the ratio of triglyceride to the total lane intensity was calculated. Data from three independent experiments were pooled for statistical analysis.

### Assessment of fatty acid transfer from adipocytes to bacteria.

Differentiated adipocytes were labeled by treatment with 1 μCi sodium [^14^C]acetate (catalog no. ARC0173) or [^14^C]oleic acid (catalog no. ARC0297) (American Radiolabeled Chemicals, Inc.) for 16 h. Extra label was removed after 16 h by washing the cells three times with medium. After the monolayer was washed with medium, cells were infected at an MOI of 1 for 24 h. Cells were harvested at day 10 after infection to obtain lipid from bacteria isolated from infected cells as mentioned above.

### Construction of autoluminescent mycobacteria.

The codon-optimized *luxCDABE* operon of the P. luminescens *lux* genes from pMU1 (24) was cut with NdeI and EcoRI and was ligated into the mycobacterial expression vector pTC-0X1L (25) digested with same enzymes to yield pTC-GCLux. Transformants of H37Rv/pTC-GCLux, referred to as H37Rv/GC-lux, were used for cellular infections.

### Estimation of iron.

Iron was estimated by the ICP-MS method. Eight million preadipocytes were either left undifferentiated or differentiated to adipocytes. Cells were washed with PBS, followed by trypsinization. The trypsinized cells were spun down at 800 rpm, washed once in PBS, and resuspended in PBS. The pellet was harvested by baking in nitric acid and hydrogen peroxide before being subjected to ICP-MS at Arbro Pharmaceuticals Pvt. Ltd.

### Estimation of ferritin.

Ferritin immunoblotting was performed from lysates of 3T3L1 preadipocytes and adipocytes and lysates of RAW 264.7 cells that were incubated with or without 200 μM oleic acid (catalog no. O3008; Sigma) treatment for 48 h. Lysates were prepared in 0.3% 3-[(3-cholamidopropyl)-dimethylammonio]-1-propanesulfonate (CHAPS) buffer; 40 μg protein from each sample was probed for the level of ferritin using sodium dodecyl sulfate-polyacrylamide gel electrophoresis (SDS-PAGE), followed by immunoblotting using an anti-ferritin antibody (ab75973; Abcam). Anti-α-tubulin (CST-11H10; Cell Signaling Technology) and anti-β-actin (ab8226; Abcam) were used as housekeeping controls for 3T3L1 and RAW 264.7 cells, respectively. Detection was performed using fluorophore-conjugated secondary antibodies scanned using a Li-Cor Odyssey system. Quantification was done using the raw integrated intensity of the ferritin signal with the α-tubulin signal.

### Measurement of the cytosolic redox potential of intracellular M. tuberculosis.

A total of 2 × 10^5^ adipocytes and preadipocytes were seeded in a 96-well black plate and were infected at an MOI of 1 for 24 h with a plasmid expressing pMrx1-roGFP2. Ten days postinfection, the infected cells were washed once in PBS, and a fluorescence reading for emission at 520 nm was taken by excitation at 390 nm and 490 nm. For oxidizing or reducing, the cells were treated with 10 mM cumene hydroperoxide (CHP) or 40 mM DTT for 15 min, and then a fluorescence reading was taken using a Tecan M200 Pro reader. A log-phase culture grown in 7H9 complete medium *in vitro* was taken as the control; it was washed once in PBS, and after resuspension of the pellet in PBS, 200 μl was transferred to the black plate in triplicate for fluorescence reading. Bacterial samples were either left untreated or treated with 10 mM CHP or 40 mM DTT for full oxidization or reduction in order to calculate the E_MSH_ value for this *in vitro*-grown culture or for Mtb^A^ and Mtb^P^ as described in reference 32.

### Checking for the sensitivity of M. tuberculosis to oxidizing agents.

A total of 0.25 × 10^6^ adipocytes and preadipocytes were seeded in a 48-well plate and were infected with H37Rv at an MOI of 1 for 24 h. Ten days postinfection, the infected cells were lysed in distilled water containing 0.1% Triton X-100 (Sigma) for preadipocytes and 1% Triton X-100 for adipocytes, and lysates were plated at various dilutions on 7H10 agar supplemented with OADC or OADS containing different concentrations of oxidizing agents, such as 0.1 and 0.01 mM cumene hydroperoxide or 0.01 and 0.02 mM plumbagin.

### Mycobacterial growth assay in different media.

H37Rv and the Δ*bfrAB* mutant were grown in 7H9 complete medium until log phase. Then the cultures were spun down and were washed twice in 7H9 medium only. The pellet was resuspended in 7H9 medium only and was inoculated into different media at an optical density (OD) of 0.05. Cultures were grown in triplicate in a 96-well plate, and the absorbance at 600 nm was taken on a daily basis. The composition of different carbon source media was as follows: 7H9 medium, 0.5% fatty-acid-free BSA (catalog no. A7030; Sigma), 0.085% sodium chloride (Sigma), and 0.025% tyloxapol (Sigma), with sole carbon sources or a mixture of 0.2% glucose (HiMedia), 0.5% glycerol (HiMedia), and oleic acid (0.09 mM, 0.18 mM, or 0.36 mM; Sigma).

For the growth assay in the presence or absence of ferritin (catalog no. F4503; Sigma), different concentrations of ferritin (1 ng, 100 ng, 1 μg/ml, 100 μg/ml) were added to a medium with glycerol as the sole carbon source. Apoferritin (catalog no. A3660; Sigma) was inactivated by heating at 95°C for 5 min. Apoferritin and heat-inactivated apoferritin were added at different concentrations (1 ng, 100 ng, 1 μg/ml, 100 μg/ml) to a medium with glycerol as the sole carbon source.

### Measurement of the cytosolic redox potential of H37Rv grown in different media.

Mrx1-roGFP2 expressing H37Rv was grown until log phase in 7H9 complete medium and then, after two washes in 7H9 medium only, was inoculated into different media at an OD of 0.05. After 6 days of growth, the cultures were washed once in PBS and resuspended in PBS. Portions (200 μl) of these cultures were taken in triplicate in a black plate for fluorescence reading as described above. Bacteria grown in 7H9 complete medium containing 0.18 mM oleic acid, 0.5% glycerol, 0.2% dextrose, 0.5% fatty-acid-free BSA, and 0.025% tyloxapol were treated with 10 mM CHP or 120 mM DTT to fully oxidize or reduce the bacterial cytosol so as to calculate E_MSH_ for bacilli grown in different media.

### Mouse infection and immunostaining.

C3HeB/FeJ mice (stock 000658; The Jackson Laboratory) were infected by the aerosol route to achieve 50 CFU/lung using a Glas-Col inhalation exposure system. Mice were euthanized at day 49, and lungs were harvested in 10% neutral buffered formalin. Sucrose-equilibrated tissues were then cryosectioned at a section depth of 5 μm using a Leica CM1850 cryostat. Cryosections were probed with anti-ferritin antibodies, followed by an Alexa Fluor 633-tagged secondary antibody. Sections were subsequently stained with Bodipy 493/503 and DAPI.

### Statistical analysis.

Statistical analyses were performed in GraphPad Prism. Statistically significant differences in triglyceride levels, luminescence, gene expression, and CFU counts across two groups were evaluated using Student's two-tailed unpaired *t* test. For the statistical analysis of RNA-seq data, see “Transcriptome profiling and differential expression analysis” above.

### Ethics statement.

The animals were housed and handled at the CSIR-Institute of Genomics and Integrative Biology, New Delhi, India, according to the directives and guidelines of the Committee for the Purpose of Control and Supervision of Experiments on Animals (CPCSEA). This work was approved by the CPCSEA as per ethics proposal IGIB/IAEC/36/37.

### Accession number(s).

All raw data files from RNA sequencing are available at NCBI GEO under accession no. GSE93362.

## Supplementary Material

Supplemental material
