# Supplementary material for: Adipocyte Model of Mycobacterium tuberculosis Infection Reveals Differential Availability of Iron to Bacilli in the Lipid-Rich Caseous Environment
Source: Infect Immun. 2018 May 22;86(6):e00041-18. doi: 10.1128/IAI.00041-18 (PMC5964510; doi:10.1128/IAI.00041-18)
Supplement: Supplemental material [file supp_86_6_e00041-18__index.html]

Supplemental material 

# Adipocyte Model of Mycobacterium tuberculosis Infection Reveals Differential Availability of Iron to Bacilli in the Lipid-Rich Caseous Environment

## Supplemental material

- Supplemental file 1 -

  Supplemental file 1. RNA-seq data.

  XLSX, 1.7M
- Supplemental file 2 -

  Supplemental file 2. RNA-seq data.

  XLSX, 36K
- Supplemental file 3 -

  Supplemental file 3. Comparison with Rodriguez et al.

  XLSX, 16K
- Supplemental file 4 -

  Supplemental file 4. Sequences of the primers used for qRT-PCR.

  PDF, 142K
- Supplemental file 5 -

  Fig. S1. (A) Thin-layer chromatogram of total lipid extract resolved for neutral lipids isolated from 3T3L1 adipocytes and preadipocytes. (B) Cellular necrosis measured by SYTOX and DAPI staining of infected cells. Fig. S2. Transcript abundance of *fas* normalized to that of the 16S rRNA gene at d10 after infection with MtbA and MtbP. Fig. S3. (A) Transcript abundance of 20 putative triglyceride synthases at d10 after infection with MtbA and MtbP. (B) Expression of *tgs1* and Rv3371 at d10 after infection with MtbA and MtbP. (C) Expression of dormancy genes at d10 after infection with MtbA and MtbP. Fig. S4. Circos image representing transcriptional network of differentially expressed genes of *M. tuberculosis* in adipocytes versus preadipocytes. Fig. S5. Transcript abundance of genes of IdeR regulon at d10 after infection with MtbA and MtbP. Fig. S6. Ferritin expression in RAW 264.7 cells with and without oleic acid treatment for 48 h. Fig. S7. EMSH of *M. tuberculosis* grown in increasing concentrations of oleic acid. Fig. S8. Density of the Δ*bfrAB* strain normalized to that of wild-type H37Rv at d7 in the presence or absence of apoferritin.

  PDF, 709K
