# Supplementary material for: Adipocyte Model of Mycobacterium tuberculosis Infection Reveals Differential Availability of Iron to Bacilli in the Lipid-Rich Caseous Environment
Source: Infect Immun. 2018 May 22;86(6):e00041-18. doi: 10.1128/IAI.00041-18 (PMC5964510; doi:10.1128/IAI.00041-18)
Supplement: Supplemental material [file IAI.00041-18_zii999092431s4.pdf]

Sequences of the primers used for qRT-PCR:

bfrA_forward: CATCGGTTCGTTGCGTATCG

bfrA_reverse: CTGGTGGTGTCCTGTTTCTC

bfrB_forward: GCGGCACAACAATATGTCGC

bfrB_reverse: GCACGAGCATCATTGCATGG

devR_forward: CCGATCTGCGCTGTCTGATC

devR_reverse: GTCCAGCGCCCACATCTTT

fas_forward:ACCTGCTGCTGGCTACCTAC

fas_reverse:CGACGTGGTGGATTCCTTGG

irtB_forward: TCCCGCGTATCGAGTTCGAC

irtB_reverse: TTCCCGGCTGCAAGCAGAAG

mbtA_forward: CTGCGGTTGTTGCAGGTTGG

mbtA_reverse: CCGATGCGGGTGAAGTTCAG

mbtD_forward: CCGCCGTCGTTGACAACTTC

mbtD_reverse: CACAGCAGGGTTCGCACATC

narK2_forward: GACCTGGGAGATGTCGTTTC

narK2_reverse: TGATGTAGGTGGGCAGGTAG

narX_forward: ATGATGGGCGAACTCTTCTG

narX_reverse: CAGCCGAATTTGTCATAGCG

Rv3371_forward:TGGTCAACGGTATCGAACTG

Rv3371_reverse: TACGCCGATCCTTGGTAAAC

tgs1_forward: CGATCCCGACTTCGATCTTG

tgs1_reverse:AGGCCTTCGATGACCCAGAC

Rv0282_forward: CGATTTCCACCTCGCGTATG

Rv0282_reverse: GATTGGCCTTGGCAAACTCC

Rv0283_forward: TCCTACGCCCTGAAAGACTC

Rv0283_reverse: CCAATACCGGCGAAATCTGC

Rv0291_forward: TCAGCGCGTCGTGTGATTCC

Rv0291_reverse: ACGCCACCGACAACACGTAG

Rv0292_forward: TTGGTGGCTGCGGATTCTCG

Rv0292_reverse: ATCGGGCAACTCGGCATTGG

16S_forward: TACGTTCCCGGGCCTTGT

16S_reverse: AATCGCCGATCCCACCTT
