# Supplementary material for: Adipocyte Model of Mycobacterium tuberculosis Infection Reveals Differential Availability of Iron to Bacilli in the Lipid-Rich Caseous Environment
Source: Infect Immun. 2018 May 22;86(6):e00041-18. doi: 10.1128/IAI.00041-18 (PMC5964510; doi:10.1128/IAI.00041-18)
Supplement: Supplemental material [file IAI.00041-18_zii999092431s5.pdf]

Figure S1

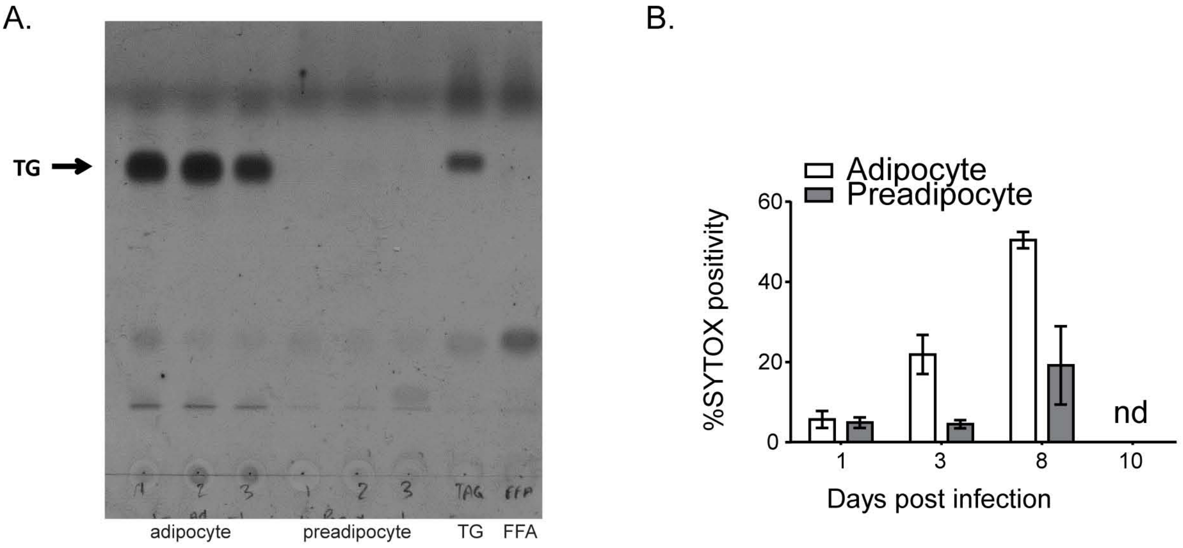

Figure S1. (A) Thin layer chromatogram of total lipid extract resolved for neutral lipids isolated from 3T3L1 adipocytes and preadipocytes. TG and FFA indiacte triglyceride (triolein) and free fatty acid (oleic acid) as standards. Triplicate wells of each cell type were analyzed. (B) Cellular necrosis measured by SYTOX and DAPI staining of infected cells. SYTOX positivity could not be determined at d10 as very few DAPI positive nuclei could be counted. Bars represent mean ± sd, n=3 wells from a representative experiment.

Figure S2

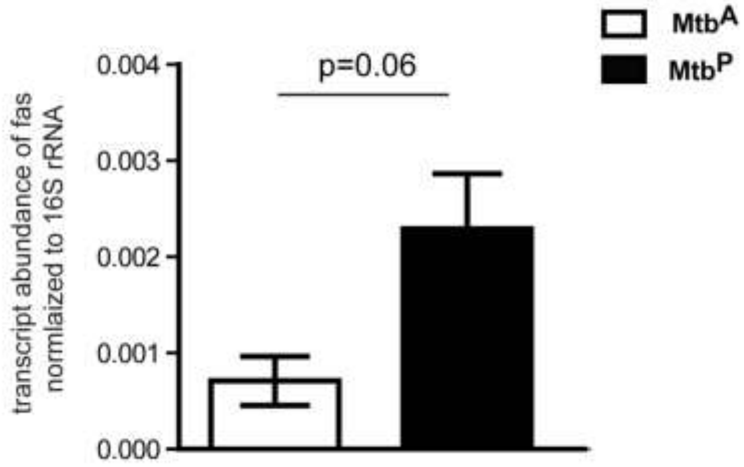

Figure S2. Transcript abundance of *fas* normlaized to that of *16S rRNA* at d10 post infection from Mtb^A and Mtb^P. Data represent mean ± se,m from 3 independent experiments.

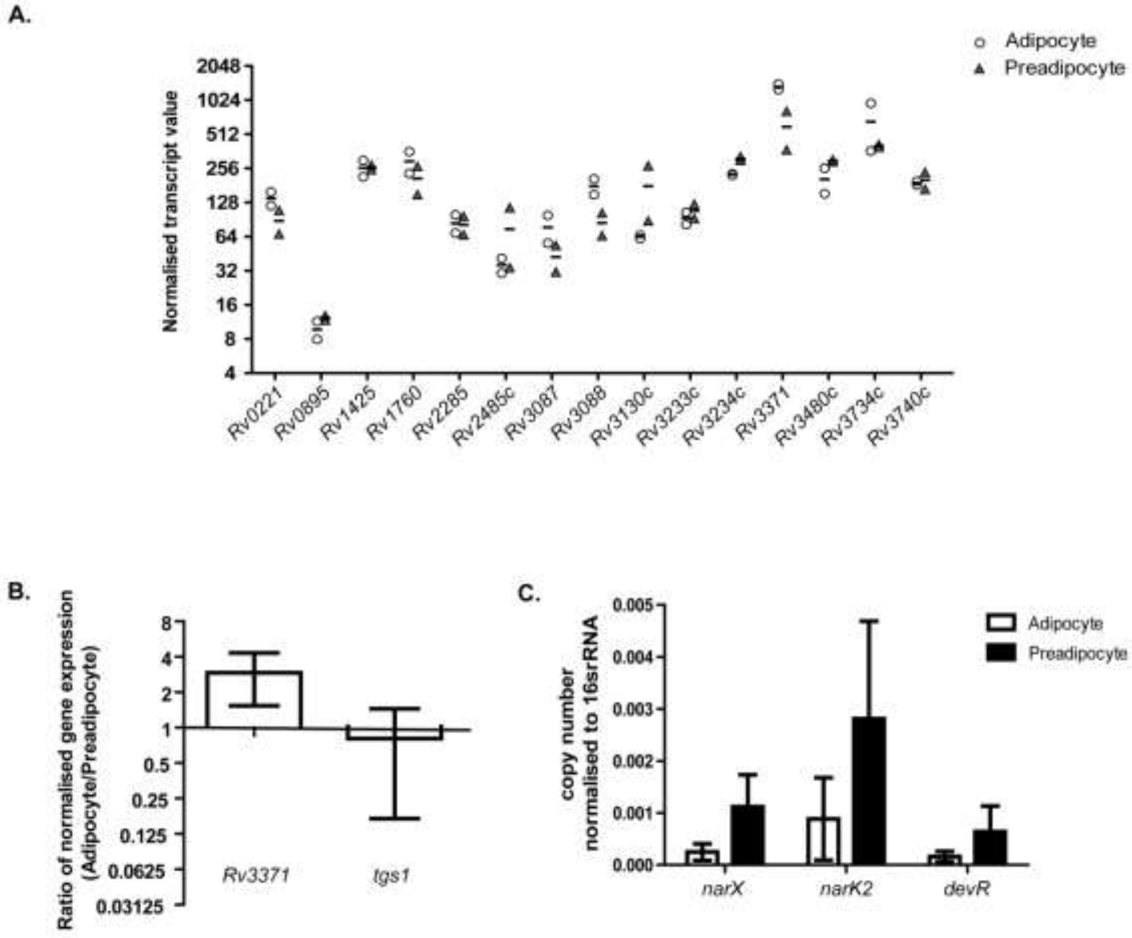

Figure S3. (A) Transcript abundance of 20 putative triglyceride synthases at d10 post infection from Mtb[A] and Mtb[P]. (B) Expression of tgs1 and Rv3371 at d10 post infection from Mtb[A] and Mtb[P]. (C) Expression of dormancy genes at d10 post infection from Mtb[A] and Mtb[P].

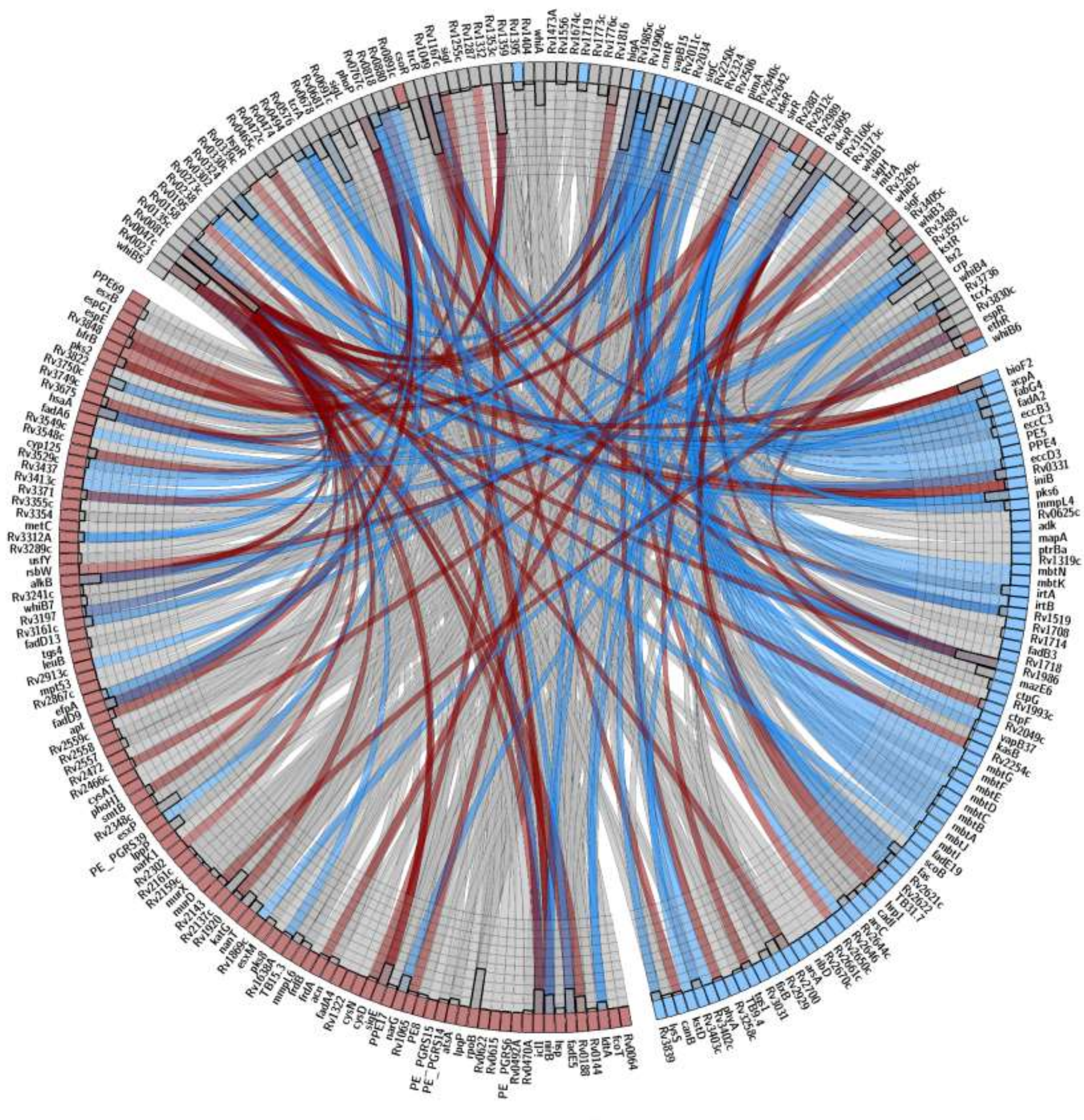

Figure S4

Circos image representing transcriptional network of differentially expressed genes of *M. tuberculosis* in adipocytes versus preadipocytes.

Boxes in the outer ring (ideogram) represent each gene in our data (fill color: grey - non-DE, red - sig up, blue - sig down). Genes are divided into 3 clusters, the cluster from 10 o' clock to 2 o' clock are Transcription Factors, 2-6 and 6-10 o' clock clusters represent over and under-expressing target genes, respectively. Transcriptional regulation of TF to their target genes are shown as ribbon emanating from the TF cluster and ending in the target clusters, ribbon colors red and blue indicate inductive and repressive regulation whereas grey ribbons show unknown regulation type. Additionally, there is a bar plot showing degree of connections for each gene.

Figure S5

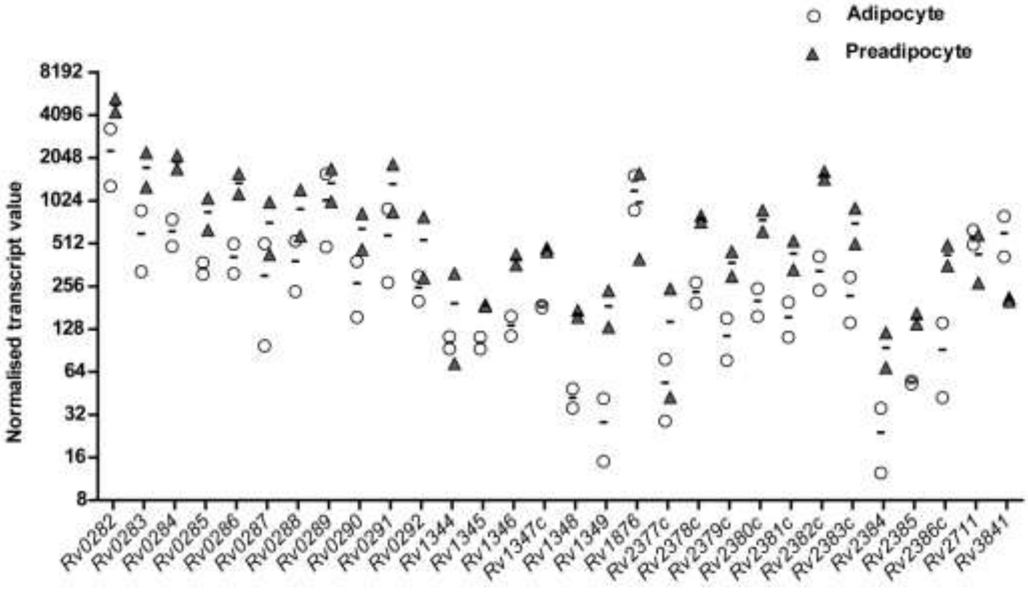

Transcript abundance of genes of IdeR regulon at d10 post infection from Mtb^{A} and Mtb^{P}.

Figure S6

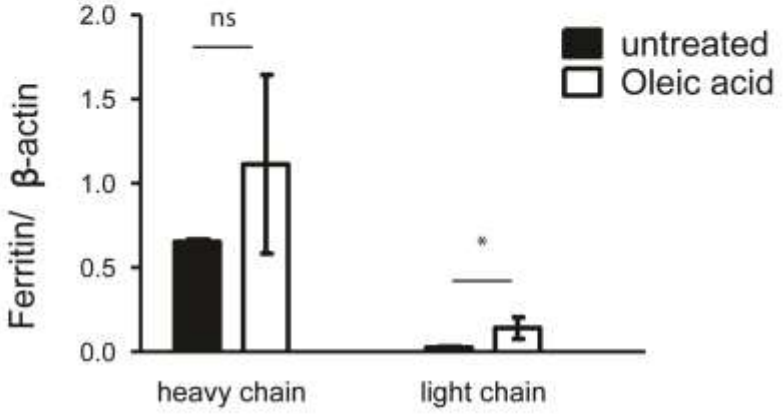

Ferritin expression in RAW264.7 cells with and without Oleic acid treatment for 48 hours.*$p<0.05$.

Figure S7

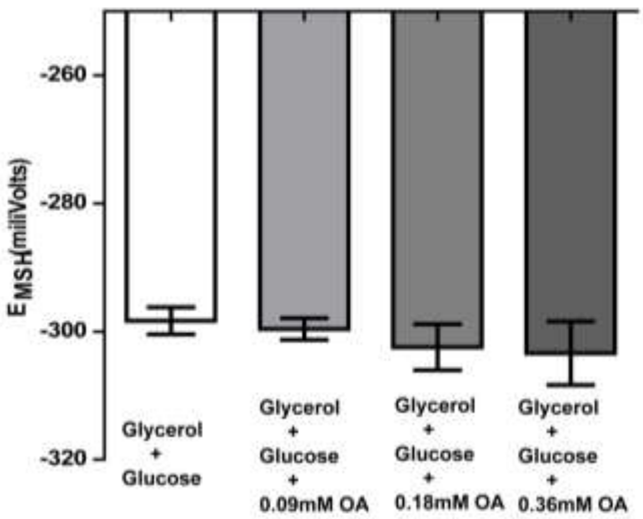

EMSH of Mtb grown in increasing concentrations of oleic acid.

Figure S8

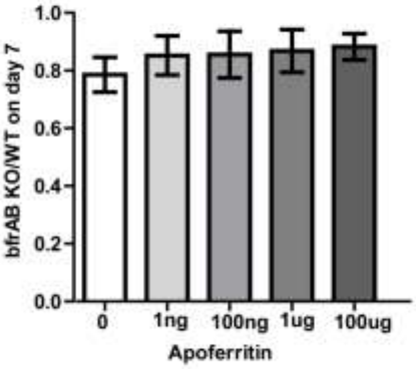

Density of ΔbfrAB normalized to that of wild type H37Rv at d7 in presence or absence of apoferritin.
